# Editorial: Zoonotic Diseases: Their Host and Vectors

**DOI:** 10.3389/fvets.2021.773151

**Published:** 2021-10-25

**Authors:** Rodrigo Morchón, Rubén Bueno-Marí, Laura Rinaldi, Elena Carretón

**Affiliations:** ^1^Zoonotic Disease and One Heatlh Group, Faculty of Pharmacy, Campus Miguel Unamuno, University of Salamanca, Salamanca, Spain; ^2^Laboratorios Lokímica, Departamento de Investigación y Desarrollo (I+D), Valencia, Spain; ^3^Área de Parasitología, Departamento de Farmacia y Tecnologia Farmacéutica y Parasitología, Facultad de Farmacia, Universitat de València, València, Spain; ^4^Department of Veterinary Medicine and Animal Production, University of Naples Federico II, Naples, Italy; ^5^Internal Medicine, Faculty of Veterinary Medicine, Research Institute of Biomedical and Health Sciences (IUIBS), University of Las Palmas de Gran Canaria, Las Palmas de Gran Canaria, Spain

**Keywords:** zoonotic disease, vectors, host, one health, reservoirs

When Frontiers in Veterinary Science asked us to produce a Research Topic, we were aware of the importance and dissemination it could have, so we tried to address an interesting, attractive, and practical topic that would be of help to the scientific community and the general public. Themes dealing with the One *Health* concept and zoonotic diseases are on the rise. The One *Health* concept, involving collaboration between veterinary and medical scientists, policy makers, and public health officials, is necessary to foster joint cooperation and control of emerging zoonotic diseases. Zoonotic diseases, which are caused by a wide range of arthropods, helminths, protozoa, bacteria and viruses, can cause severe and even fatal clinical conditions in animals and seriously affect the infected humans. The main zoonoses are related to interactions between livestock and wildlife, as well as between dogs and cats and human populations. Humans are accidentally infected in endemic areas, where animals act as *reservoirs* and climatic conditions favor the proliferation of vectors. The influence of other variables, such as temperature, humidity, presence of irrigated areas, introduction of new vector species, climate change, increasing human activity, travel with pets to/from endemic countries and the presence of these diseases in areas previously not described as endemic, are important factors to consider in the establishment of new zoonotic diseases in areas where, until then, were considered free of the disease. Approximately 60% of human diseases are zoonotic and at least 75% of the emerging pathogens of human infections are of animal origin. Currently, most of these diseases are neglected despite causing a potentially global problem.

Therefore, this Research Topic entitled *Zoonotic Diseases: Their Host and Vectors* was proposed with the aim of providing state-of-the-art research focused on preventing and controlling zoonotic diseases, both through the control of the vectors and their animal reservoirs. It contains a total of 16 contributions from parasitologists, immunologists, entomologists, veterinarians, virologists, and microbiologists from all continents, who have addressed the study of different zoonotic diseases, dealing with topics such as the relationship between the human population, domestic animals and wildlife, the role of invasive alien species, the epidemiology of zoonotic infections, different strategies in the monitoring and control, programmes for treatment and prevention, vector dynamics, vector life cycles, and immune response in their hosts.

## Author Contributions

RM, RB-M, LR, and EC wrote the editorial. All authors listed have made a substantial, direct and intellectual contribution to the work, and approved it for publication.

## Conflict of Interest

The authors declare that the research was conducted in the absence of any commercial or financial relationships that could be construed as a potential conflict of interest.

## Publisher's Note

All claims expressed in this article are solely those of the authors and do not necessarily represent those of their affiliated organizations, or those of the publisher, the editors and the reviewers. Any product that may be evaluated in this article, or claim that may be made by its manufacturer, is not guaranteed or endorsed by the publisher.

